# Failure of Arm Movement Control in Stroke Patients, Characterized by Loss of Complexity

**DOI:** 10.1371/journal.pone.0141996

**Published:** 2015-11-04

**Authors:** Segun Goh, Kyungreem Han, Jehkwang Ryu, Seonjin Kim, MooYoung Choi

**Affiliations:** 1 Department of Physics and Astronomy and Center for Theoretical Physics, Seoul National University, Seoul 151–747, Korea; 2 Institute for Cognitive Science, College of Humanities, Seoul National University, Seoul 151–742, Korea; 3 Department of Physical Education, Seoul National University, Seoul 151–748, Korea; Tianjin University, CHINA

## Abstract

We study the mechanism of human arm-posture control by means of nonlinear dynamics and quantitative time series analysis methods. Utilizing linear and nonlinear measures in combination, we find that pathological tremors emerge in patient dynamics and serve as a main feature discriminating between normal and patient groups. The deterministic structure accompanied with loss of complexity inherent in the tremor dynamics is also revealed. To probe the underlying mechanism of the arm-posture dynamics, we further analyze the coupling patterns between joints and components, and discuss their roles in breaking of the organization structure. As a result, we elucidate the mechanisms in the arm-posture dynamics of normal subjects responding to the gravitational force and for the reduction of the dynamic degrees of freedom in the patient dynamics. This study provides an integrated framework for the origin of the loss of complexity in the dynamics of patients as well as the coupling structure in the arm-posture dynamics.

## Introduction

Since the pioneering study of the phase transition emerging in human hand movements [1], dynamics of human body movements has attracted much attention of physicists equipped with dynamical systems theory and nonlinear dynamics. Widely studied topics include intermittency [2] and criticality [3, 4] as well as the roles of sensory time delay [5, 6]. Nonlinear time series analysis [7, 8] is also proved to be a useful method applied to various bio-signals including human body movements, albeit still in its infancy. In particular, whether the deterministic chaos is inherent in the motor system and assessable from the human body movement time series is an extensively discussed subject [9, 10]. On the other hand, there was also an attempt to understand the human body movement as a correlated random walk [11], which led to the stochastic process modeling [12, 13]. It is thus regarded that nonlinearity, time delay, and randomness are crucial components in the complex dynamics of human movements.

Among various human movements, postural sway dynamics in quiet standing has been studied extensively via time series analysis and modeling. While the surrogate data method has confirmed that the center-of-pressure trajectory is characterized by a correlated noise [11], nonlinear dynamics measures such as entropy are still considered to be promising for evaluating posture controls related to injuries [14]. As for the essential dynamics of postural sway, the most well-known framework is the inverted pendulum model [15]. Another representative example of human movements is the planar arm movement [16], which has been described by, e.g., the minimum jerk model [17] and the minimum torque-change model [18].

There are also studies of the goal-directed arm-posture task [19]. Related to the task, most widely discussed topic is the physiological and pathological tremors [20], which provide a unique characteristic in arm-posture dynamics. In fact, controlling/minimizing the tremors is directly related to the performance of target shooting [21] or microsurgery [22]. Therefore the arm-posture and tremor dynamics has been extensively studied by means of linear and nonlinear measures [23–27]. Due to its intrinsic complex nature, however, relatively little attention has been paid to the essential dynamics of the arm-posture task and the emergence of tremors, compared with postural sway or planar arm movement dynamics.

In this paper, we thus consider goal-directed arm-posture dynamics and analyze time series measured from normal control subjects and stroke patients [28], focusing on the underlying mechanism for the human motor system to respond to the environment and to retain dynamical complexity. The unique characteristics of the normal arm-posture dynamics which this study deals with are summarized as follows: First, it is in general aperiodic and distinct from the dynamics of the gait [29] or hand movements [1]. Second, cooperation between joints is tangible. Although postural sway dynamics is also accomplished by such cooperation between various parts of the human body [30, 31], the joints in the arm are much more clearly segmented and seem to perform in a highly organized fashion. Moreover, there exists an external factor, the gravitational force, which perturbs and compels the system to react to it. Note that the gravitational force affects the system asymmetrically in the arm-posture task, in contrast to the postural sway dynamics. Analyzing the time series by means of various quantitative measures, we address these issues and suggest an integrated framework in which essential dynamics of the arm posture is described.

## Results and Discussion

### Spectral Analysis and Pathological Tremors

**Fig 1:**
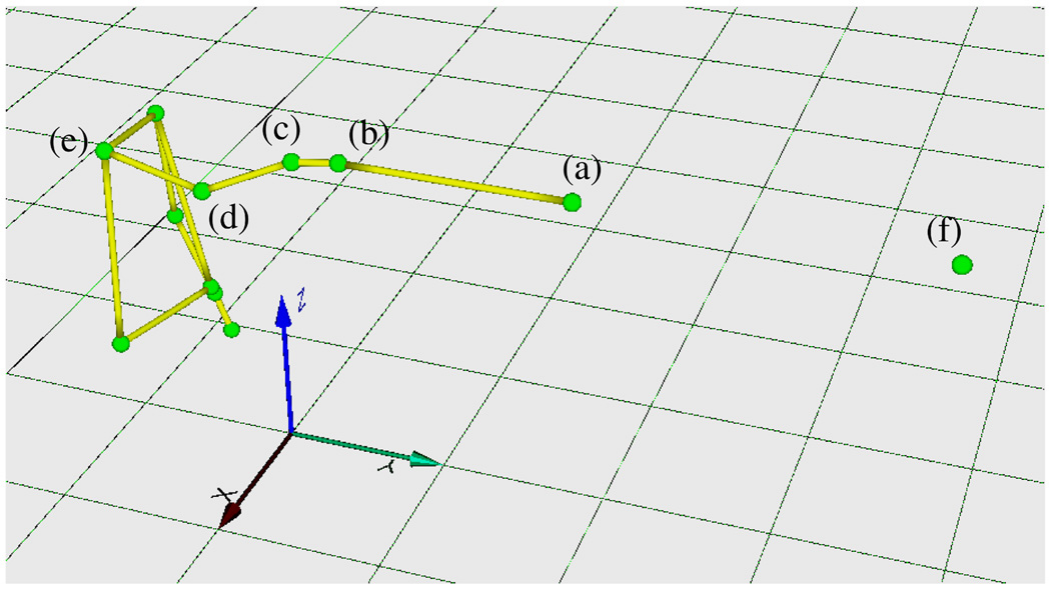
Snapshot of the arm-posture task and directions of *X*-, *Y*- and *Z*-axes in the Cartesian coordinate system. Green dots represent the location of the attached markers: (a) the end of the rod $r_{\alpha }\equiv \left\{s_{\alpha }^{1}\left(t\right)\right\}$, (b) the finger $f_{\alpha }\equiv \left\{s_{\alpha }^{2}\left(t\right)\right\}$, (c) the wrist $w_{\alpha }\equiv \left\{s_{\alpha }^{3}\left(t\right)\right\}$, (d) the elbow $e_{\alpha }\equiv \left\{s_{\alpha }^{4}\left(t\right)\right\}$, and (e) the shoulder $s_{\alpha }\equiv \left\{s_{\alpha }^{5}\left(t\right)\right\}$. The dot (f) indicates the location of the target.

There are five multivariate time series available, describing dynamics of the pointing rod, finger, wrist, elbow, and shoulder joints (hereafter, for convenience of notation, the end of the pointing rod is also considered as a joint), where markers are attached. Each time series consists of three components in the *X*-, *Y*-, and *Z*-directions. The coordinate system is illustrated in Fig 1: The *Z*-axis is taken to be vertical whereas the *Y*-axis to be directed toward the target. Note that the main goal of the arm-posture task is to maintain the rod in the *Y*-direction, i.e., to keep pointing the rod at the target. Therefore the dynamics in the *Y*-direction is irrelevant to the task. We represent time series of the *X*-, *Y*- and *Z*-components of each joint as $s_{\alpha }^{i}\left(t\right)$ (with *s* ^*i*^ = *r*, *f*, *w*, *e*, *s* for *i* = 1, ⋯, 5, respectively, and *α* = *X*, *Y*, *Z*), where *r*, *f*, *w*, *e*, and *s* denote the end of the rod, finger, wrist, elbow, and shoulder joints, respectively.

**Fig 2:**
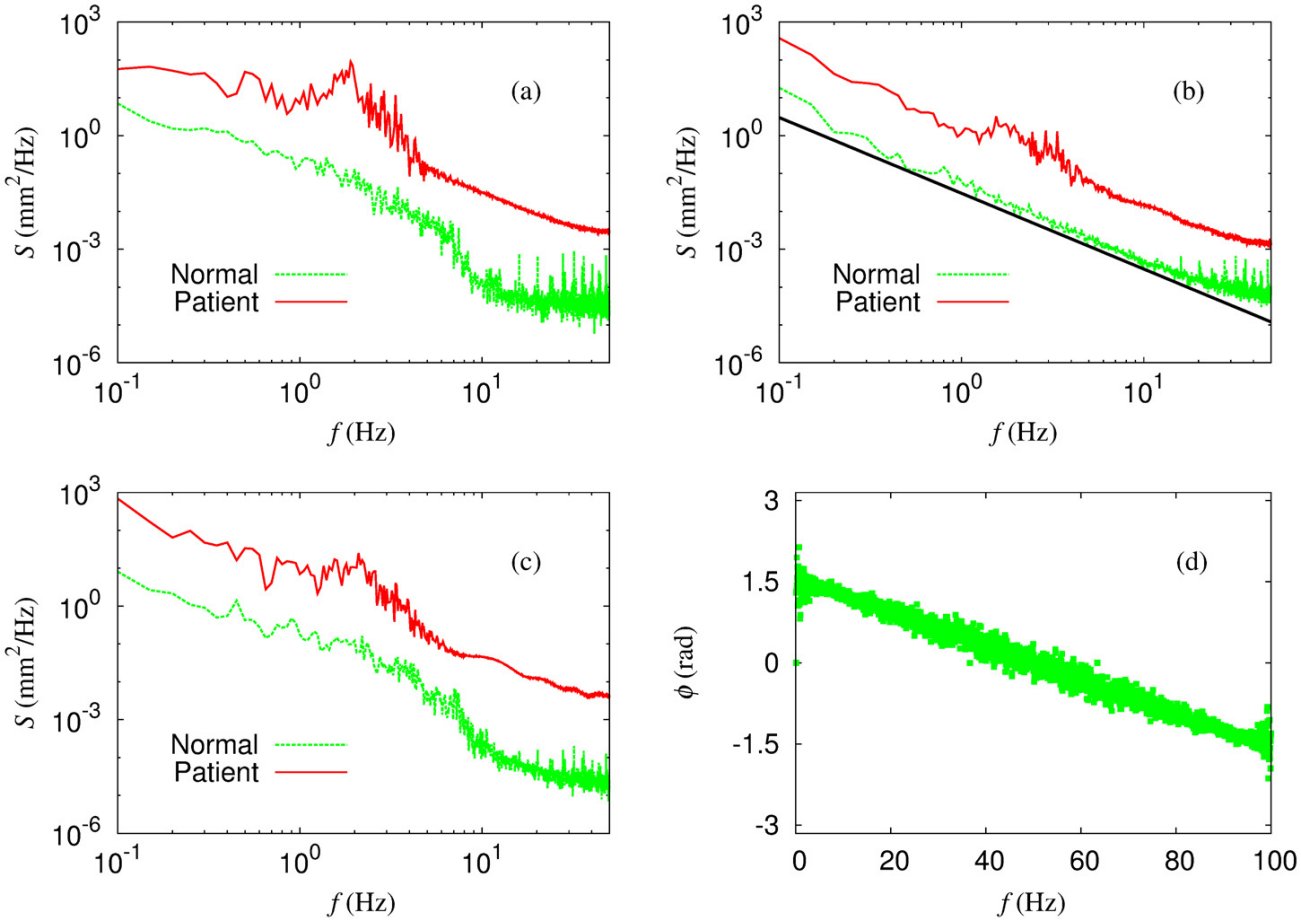
Average power spectrum of the time series of the position of the rod end. (a) *X*-component, (b) *Y*-component, and (c) *Z*-component, measured in the normal subject group (green dotted lines) and in the patient group (red solid lines). The straight line in (b), having the slope two, serves as a guide to the eye. In (d) the phase spectrum of the time series *r*_*Y*_ of normal subject #1 is plotted.

Examining the time series carefully, we recognize a substantial difference in the time series between normal subjects and patients: The time series of the normal group are contaminated by noise-like signals, notably in the high-frequency regime, even though they may not be the manifestation of actual noises as discussed later. In contrast, those of the patient group appear more or less smooth. To quantify the difference, we first compute the power and phase spectra of the arm-posture time series, utilizing the fast Fourier transform (FFT) algorithm, and plot the results of the rod joint in Fig 2. It is observed that the power spectrum of the patient time series exhibits peaks around 2 to 3 Hz; this suggests the oscillatory dynamics to be interpreted possibly as the pathological tremor [32]. Note that similar patterns are also observed in the data for other joints. Although the overall power-law behavior of the power spectrum tends to obscure the period of the motion, one can expect that there might exist deterministic, low-dimensional chaotic structure in the essential dynamics of the patients. We thus analyze the time series in detail with the aid of the nonlinear time series analysis, to which the next section is devoted.

In addition, the 1/*f* ^2^ power spectrum, which is an indicator of the Brownian motion, is observed in the *Y*-component time series of normal subjects, as shown in Fig 2b. On the other hand, Fig 2d discloses rather an aligned structure of the phase spectrum. Simulating a random walk and analyzing the corresponding time series, we observe similar but more scattered structure in the phase spectrum. We thus presume that the *Y*-component dynamics is distinct from a pure random walk or Brownian noise.

Henceforth we use 0.5 Hz high-pass filtered time series to reduce the effects of low-frequency dynamics, for we wish to focus on tremor dynamics. Even though the low-frequency components have larger amplitudes, they may suffer from non-stationarity and disturb inspection of the fine structure of the dynamics.

### Complexity of arm-posture dynamics

**Fig 3:**
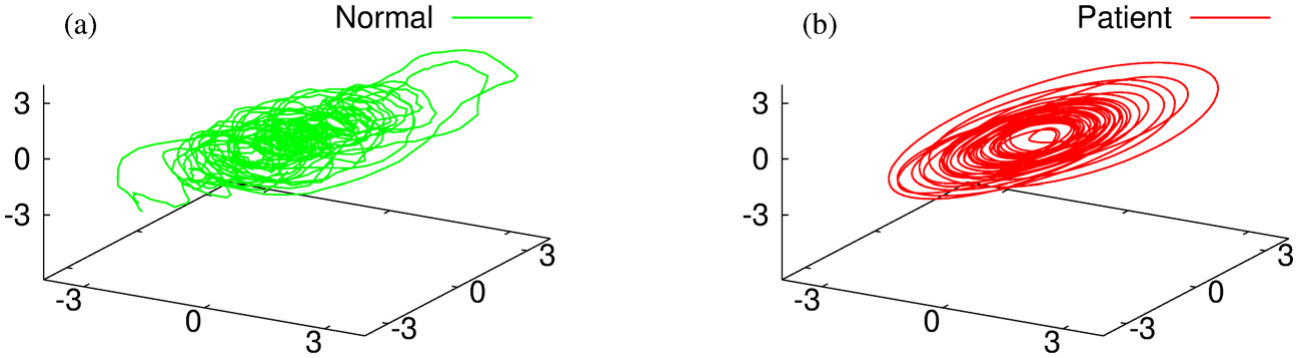
Reconstructed attractors. Typical examples from the time series of (a) normal subject #1 and (b) patient #1 are shown. Embedding dimension *m* = 26 and time delay *τ* = 5 have been used.

We now make a comparison of the complexity between the normal subject group and the patient one, based on nonlinear dynamic measures such as the dimensional complexity [33] and the multiscale entropy [34]. To compute those nonlinear measures, one should first reconstruct the attractor embedded in the phase space. Here we adopt the Takens delay embedding method [35] to obtain the reconstructed attractors, typical examples of which are shown in Fig 3. A short description of the nonlinear time series analysis measures considered in this paper is given in “Materials and Methods: Nonlinear Time Series Analysis” section. Specifically, we focus on the time series of the rod, because the definitive performance of the task should be assessed by the dynamics of the end point of the rod.

#### Dimensional Complexity

In fact to define the complexity of a system is on the cutting edge of complex system science and still remains controversial. Here we use the dimensional complexity measure [33], which is a revised version of the classical correlation dimension [36], as the complexity index in a naive manner. However, as well known, spurious values can be obtained from the time series generated by a stationary linear stochastic process. It is thus necessary to carry out the surrogate test, a standard method guaranteeing that the computed values of a nonlinear measure are consequences of determinism inherent in the dynamics [37]. To verify the deterministic structure of the time series, we employ the iterative amplitude adjusted Fourier transform (IAAFT) surrogate method [38], which provides the exact amplitude distribution as well as an almost exact power spectrum, and generate 100 surrogate data for each bare time series.

We also adopt the automatic extraction algorithm [39] to avoid the intervention of subjectivity, when determining plateaus from the plot of ∂ ln *C*/∂*ϵ* versus ln *ϵ*, where *ϵ* denotes the threshold distance. The plateau extraction procedure is as follows: There are two parameters, *length* and *unevenness*, quantifying the quality of the extracted plateau. The length is defined by the number of data points in the plateau and the unevenness the difference between the maximum and minimum values of ∂ ln *C*(*ϵ*)/ln *ϵ*, normalized by the maximum. We expect the length of the extracted plateau to be long enough but the unevenness to remain small. Here it is obvious that the unevenness of a plateau in general reduces as the length is decreased. We therefore examine the unevenness as well as the location of the extracted plateau and the dimensional complexity *D*_2_, varying the length from the longest one, to confirm the existence of a convincing plateau along with appropriate criteria. Fortunately, consistent results are obtained over a wide range of the length scale where the unevenness as well as *D*_2_ and the position of the plateau remains stable. It is thus concluded that the unevenness rather than the length provides consistent criteria. Accordingly, we relax the length limitation to guarantee the plateau extraction and choose the longest one among possible plateaus whose unevenness values are smaller than 0.4. For surrogate time series, on the other hand, we consider unevenness and length values less than 0.4 and larger than 0.1, respectively and extract plateaus successively for more than 90 time series in most cases.

**Fig 4:**
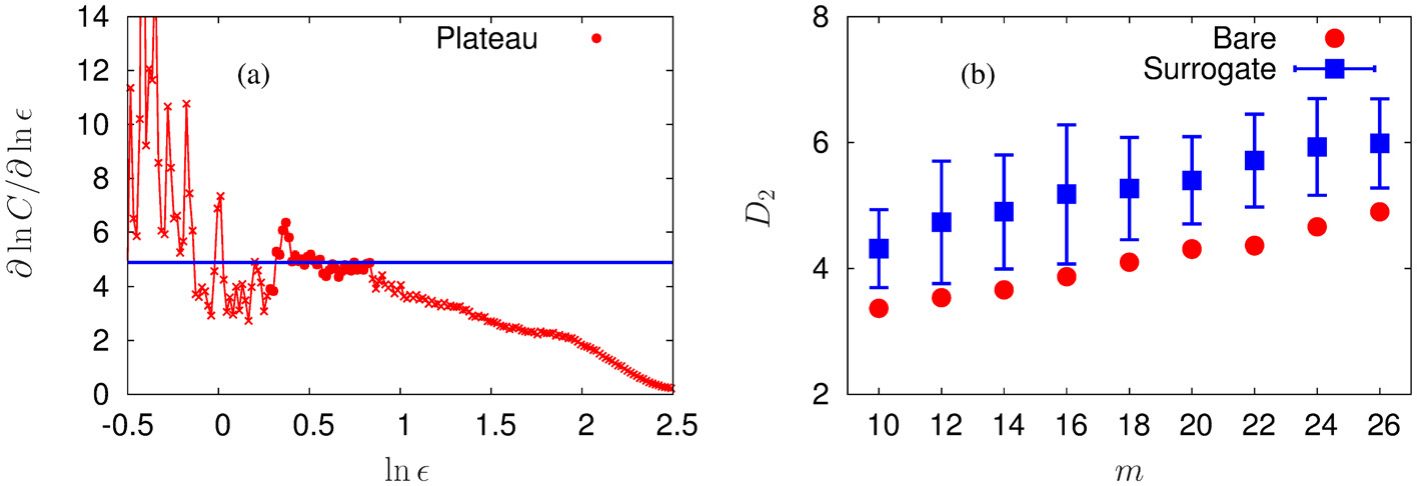
Plateau extraction and surrogate data test. (a) An example of the plateau extraction (from the *X*-component time series of patient #1). Data points labeled by red circles depict the automatically extracted plateau whereas the blue line corresponds to the dimensional complexity *D*_2_ computed from the plateau. (b) The values of *D*_2_ obtained from the bare time series (red circles) and from the surrogate time series (blue squares) are compared, with the error bars estimated by standard deviations.

Finally, Fig 4 presents, as an example, (a) the plot of ∂ ln *C*/∂*ϵ* versus ln *ϵ* for *m* = 26, with emphasis on the extracted plateau, together with (b) the comparison of the dimensional complexity *D*_2_ obtained from the bare and surrogate time series of the *X*-component of patient #1. Embedding dimensions *m* = 10, 12, 14, ⋯, 26 have been employed in the computation.

Note here that there are limitation issues encountered in interpreting the results of *D*_2_. First, we fail to determine *D*_2_ for six bare time series (*Y*-components of normal subjects #1 and #5, *Z*-components of normal subjects #4 and #6, *X*-component of patient #2, and *Y*-component of patient #5) due to a technical reason. In these cases, detected plateaus vary as the embedding dimensions are altered. This may imply multiscale characteristics of the dynamics, which remains beyond the scope of this paper. Second, there exists a fundamental limitation in the dimensional complexity associated with the limited length *N* of the time series. Counting the number of points forming the attractor (of diameter *D*) in the regime of *ρ* ≡ *ϵ*/*D* ≪ 1 [see Eq (3)] leads to the theoretical limitation value *d*_max_ = 2 log*N*/ log (1/*ρ*) [40]. As each time series analyzed in this paper consists of *N* = 2000 points in length, the upper bound of *d*_max_ is given by 6.60 if *ρ* < 0.1 is imposed as in Ref. [40]. Interestingly, the dimensional complexity *D*_2_ for all in the normal group (but the *Y*-component of subject #1) turns out to be larger than 6.60. In contrast, in the patient group, *D*_2_ exceeding this theoretical limitation is observed only in the time series of the *Y*-component of #4 and the *Z*-components of #2, #3, and #5.

It is remarkable that all the results of normal subjects are accompanied with these imitation issues; the obtained specific values could thus be spurious. In this regard, we infer that the dynamics of the normal subjects does not correspond to low-dimensional chaos. It is conceivable that high-dimensional chaos is involved, which may not be distinguished from noise in reality (or at least in our time series). In the case of patients, some time series also suffer from the technical difficulties or the theoretical limitation. In sharp contrast to the normal subjects, however, we apply the surrogate method, to confirm low-dimensional chaos in half of the patient time series which are also free from theoretical and technical limitations (see Table 1 for detailed values of *D*_2_ from bare times series of patients). Specifically, we verify that the null hypothesis of a linear stochastic stationary process is rejected consistently with the confidence interval at the 99% level of significance. Notably, we have failed to reject the null hypothesis for every patient time series accompanied with the theoretical limitation.

**Table 1:** Dimensional complexity of the stroke patient group compared with the normal group.

Patient	*X*	*Y*	*Z*
# 1	4.895^†^	5.084^†^	5.960^†^
# 2	4.831	6.448	6.719
# 3	5.966	5.572^†^	6.734
# 4	5.657^†^	8.816	6.297^†^
# 5	6.584^†^	0.236	8.093
# 6	3.001	5.241^†^	3.904^†^
Patient Group	5.156±1.137*	5.233±2.563	6.285±1.254*
Normal Group	9.019±1.572	7.376±1.287	9.253±1.481

Comparing the results of bare time series to those of surrogate series, we confirm the low-dimensional chaos in nine time series in the patient group, which are labeled by daggers (†). If we ignore the *X*-component time series of patient #6, which suffers from the technical limitation, dynamics of each component of patients #1 and #5 turns out to be low-dimensional chaos. The two lowermost rows display the average values of the dimensional complexity, together with standard deviations. Comparing the values of the patient group with those of the normal one, we confirm that the differences are statistically significant (student’s *t*-test, *p* < 0.05) in *X*- and *Z*-components; these are labeled by asterisks(*). The embedding dimension is again given by *m* = 26.

Further, we compare the dimensional complexity of the patient group with that of the normal subject group. Due to the theoretical limitation for the normal-group time series, one may argue that the result should not be interpreted as the dimension. However, we may still regard obtained values as a practical index characterizing dynamical properties of the time series. From this point of view, we analyze the values further even though one should be cautious to give the physical meaning of the dimension. As summarized in Table 1, the difference in the dimensional complexity is statistically significant for components in the *X*- and *Z*-directions. It is thus confirmed that patients suffer the loss of complexity, compared with normal subjects.

#### Multiscale Sample Entropy

In addition to the dimensional complexity, we also consider the multiscale entropy as a complexity index. In general, the entropy, measuring irregularity and therefore predictability of the time series, does not serve as an adequate measure for complexity: Specifically, a random time series has a large value of entropy even though it is in fact not complex. In the multiscale entropy, on the other hand, effects from uncorrelated noise are reduced effectively by increasing the scale factor.

**Fig 5:**
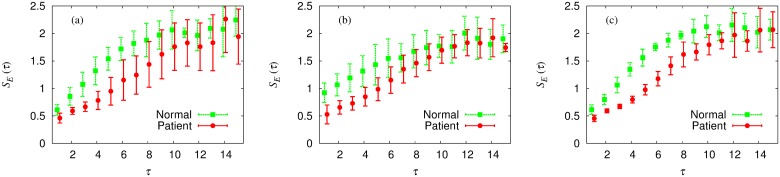
Multiscale entropy *S*_*E*_ of the time series of the rod versus the scale factor *τ*. (a) *X*-component, (b) *Y*-component, and (c) *Z*-component. Below *τ* ≈ 10, the multiscale entropy of the normal group (green) is observed to be higher than that of the patient group (red).

Fig 5 shows the results obtained from the time series of the rod. It is observed that in sharp contrast with the random case, the multiscale entropy in general increases as the scale factor *τ* is increased. This indicates that the apparent irregularity in the time series has its origin in the intrinsic rich structure rather than in simple randomness. Rather large error bars around the scale factor *τ* ≈ 10 presumably reflect the limitation on the length of the time series. Finally, comparing the entropy between the normal group and the patient group, we confirm that the time series of patients are more regular and predictable. This difference is relatively small in the case of the *Y*-component, which is consistent with the result of dimensional complexity.

### Coupling Patterns underlying arm-posture dynamics

In the previous section, we have considered the complexity of the arm-posture dynamics, which displays significant reduction in the patient group. Since the motor system is highly organized to control finely the body movement, the coupling between components may be crucial for understanding the underlying mechanism bringing complexity into the system. In this section, we thus probe the coupling between components and the organization structure of the arm-posture dynamics, by means of phase synchrony, Granger causality [41, 42], and multiscale network analysis of multivariate time series [43], to elucidate the possible origin of the impaired complexity in the patient group.

#### Phase Synchrony and Granger Causality between Joints

We first examine the coupling between joints, by computing phase synchrony. More specifically, for example, if the elbow can operate independently of other joints, the upper arm and the forearm can move irrespectively of each other and therefore the phase synchrony should be weak. On the contrary, if a certain joint is stiff, the movement of the precedent part of the joint may directly affect the following part of the arm. To probe this view, we focus on the four nearest pairs (rod-finger, finger-wrist, and so on) in the same direction. We further consider the difference $\Delta s_{\alpha }^{i}$ of the time series between adjacent joints rather than the original time series $s_{\alpha }^{i}$ of each joint, to separate the isolated dynamics of each joint. To quantify the phase synchrony, we introduce two order parameters $\Psi_{1}\left(\Delta s_{\alpha }^{i},\Delta s_{\alpha }^{i+1}\right)$ and $\Psi_{2}\left(\Delta s_{\alpha }^{i},\Delta s_{\alpha }^{i+1}\right)$, measuring in and out-of phase synchrony, respectively. The definitions of Ψ_1_ and Ψ_2_ as well as $\Delta s_{\alpha }^{i}$ are given in “Materials and Methods: Phase Synchrony” section. Note that the order parameters used in this section are defined as functions of the time series of each joint, rather than those of two adjacent joints. In other words, the order parameters serve as indices quantifying the operational independency of a certain joint from its precedent joint.

As summarized in Table 2, patients display phase synchrony significantly stronger than normal subjects for the *Y*-components of the finger and the wrist (order parameter Ψ_1_), *Z*-component of the elbow (order parameter Ψ_2_) and *Z*-component of the wrist (both Ψ_1_ and Ψ_2_). This confirms that the couplings between joints of patients are in general stronger than those of normal subjects. In the normal case, one should be able to move the joints unrestrictedly, adopting various strategies to control finely the rod. Then adjacent joints form diverse angles and directions depending on the given circumstance. On the other hand, if the joints are overly coupled due to stiffness, they cannot help moving simultaneously to perform the task. Then the time series of the adjacent joints are synchronized to each other, exhibiting enhanced phase synchrony. Interestingly, in the case of Ψ_2_, only the difference in the *Z*-components (of the wrist and the elbow joints) are significant. Structurally, these joints can also move in the out-of-phase way, and Ψ_2_ apparently reflects the inherent symmetry more precisely.

**Table 2:** Phase synchrony and Granger causality between joints.

$\Delta s_{\alpha }^{i}$	$\Psi_{1}\left(\Delta s_{\alpha }^{i},\Delta s_{\alpha }^{i+1}\right)$		$\Psi_{2}\left(\Delta s_{\alpha }^{i},\Delta s_{\alpha }^{i+1}\right)$		$G_{\Delta s_{\alpha }^{i+1}\to \Delta s_{\alpha }^{i+1}}$	
	Normal	Patient	Normal	Patient	Normal	Patient
Δ*f*_*X*_	0.45±0.09	0.49±0.21	0.16±0.09	0.28±0.23	0.19±0.11	0.16±0.13
Δ*f*_*Y*_	0.093±0.055	0.29±0.09*	0.13±0.08	0.090±0.055	0.034±0.024	0.093±0.051*
Δ*f*_*Z*_	0.43±0.07	0.53±0.17	0.15±0.07	0.31±0.17	0.13±0.16	0.20±0.04
Δ*w*_*X*_	0.30±0.09	0.44±0.17	0.075±0.042	0.22±0.14	0.046±0.017	0.040±0.024
Δ*w*_*Y*_	0.15±0.12	0.38±0.10*	0.14±0.11	0.13±0.10	0.022±0.015	0.036±0.021
Δ*w*_*Z*_	0.17±0.07	0.54±0.08*	0.07±0.04	0.30±0.09*	0.027±0.021	0.073±0.028*
Δ*e*_*X*_	0.34±0.05	0.25±0.10	0.11±0.05	0.10±0.04	0.015±0.003	0.027±0.014
Δ*e*_*Y*_	0.094±0.038	0.19±0.10	0.10±0.09	0.16±0.12	0.011±0.008	0.030±0.024
Δ*e*_*Z*_	0.36±0.03	0.44±0.11	0.10±0.04	0.21±0.08*	0.013±0.005	0.053±0.014*

Order parameter Ψ_1_ for in-phase synchrony, order parameter Ψ_2_ for out-of-phase synchrony, and Granger causality *G* between joints are summarized. Comparing the order parameters, we confirm that the *Y*-component *f*_*Y*_ of the finger, *Y*- and *Z*-components *w*_*Y*_ and *w*_*Z*_ of the wrist, and *Z*-component *e*_*Z*_ of the elbow are coupled stronger to their precedent joints in the patient group. Consistently with the phase synchrony, isolated causality $G_{\Delta s_{\alpha }^{i+1}\to \Delta s_{\alpha }^{i}}$, measured from Δ*f*_*Y*_, Δ*w*_*Z*_, Δ*e*_*Z*_, and their precedent joint (isolated) time series takes significantly larger values in the patient group. Asterisks (*) denote statistically significant normal-patient group pairs (with student’s *t*-test *p* < 0.05).

Next, we consider Granger causality between the joints, which quantifies the amount of unique information provided by a time series to forecast other series. Therefore, if the movement of a joint causes changes of another joint due to the coupling, we generally observe significant Granger causality between their time series. Here we evaluate the isolated causality measure $G_{\Delta s_{\alpha }^{i+1}\to \Delta s_{\alpha }^{i}}$ to quantify the dynamics of a certain joint, just like the phase synchrony measure $\Psi (\Delta s_{\alpha }^{i},\Delta s_{\alpha }^{i+1})$ (see the definition in “Materials and Methods: Granger Causality” section). Computing $G_{\Delta s_{\alpha }^{i+1}\to \Delta s_{\alpha }^{i}}$, we obtain results consistent with those of the phase synchrony analysis, as shown in Table 2. Namely, differences in the *Y*-component of the finger and the *Z*-components of the wrist and the elbow are statistically significant. Note that we have not considered the opposite direction, i.e., $G_{\Delta s_{\alpha }^{i}\to \Delta s_{\alpha }^{i+1}}$ in the comparison of the Granger causality. In some cases, e.g., Δ*f*_*Y*_ and Δ*r*_*Y*_ pairs in the normal subject group, the isolated causality measure of the opposite direction turns out to be larger than that of the trivial direction. This observation may indicate rather a passive/complementary role of the upper arm compared with the forearm in the arm-posture task, although detailed analysis is left for further study.

In addition, it is of interest to note that *G*(*w*_*Y*_) is not significantly larger in the patient group relative to the normal one. This seems to be in contradiction to the phase synchrony result shown in Table 2. Obviously, one may expect both the phase synchrony and the Granger causality to take large values simultaneously, if the joints are coupled to each other with the time delay inevitable in the motor system. This unexpected result turns out to be crucial in the loss of complexity, which is discussed in full in the next section.

#### Multiscale Network Analysis of Multivariate Time Series

**Fig 6:**
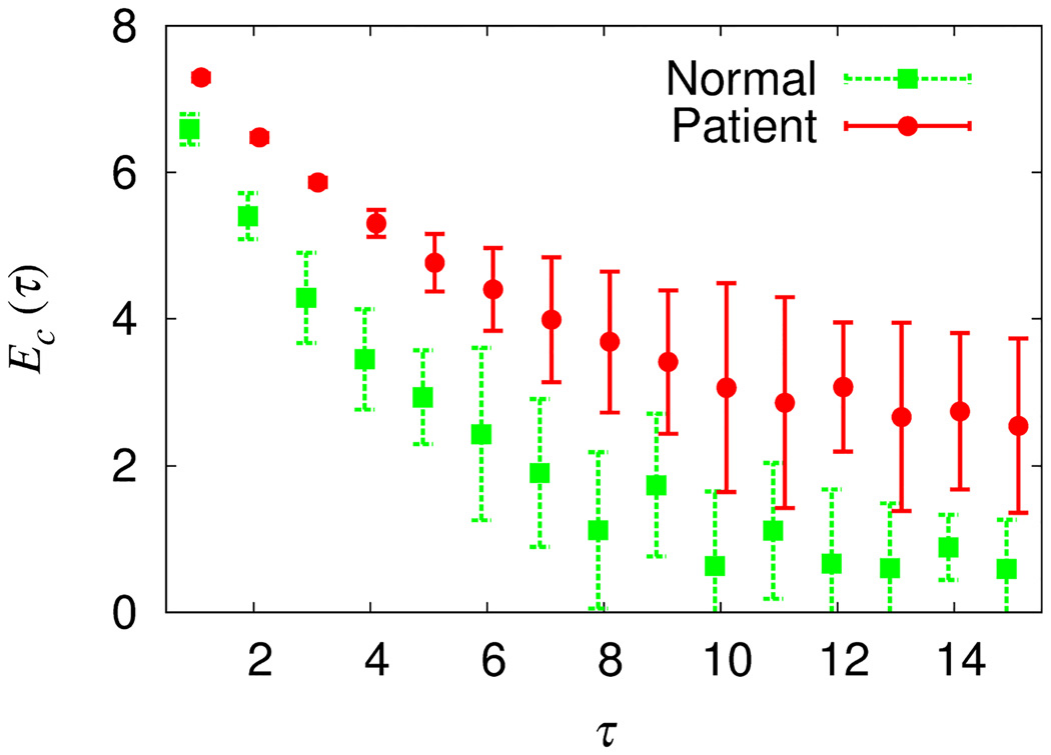
Clustering coefficient entropy *E*_*c*_ inferred from multivariate data versus scale factor *τ*. The multivariate time series consists of *Z*-components of four isolated time series. The average value of the normal group (green) is smaller than that of the patient group (red). The difference is statistically significant (student’s *t*-test, *p* < 0.05) in every examined scales (*τ* ≤ 15). Below *τ* = 5, every normal subject has a smaller value of *E*_*c*_ than every patient, as manifested by the small error bars.

Heretofore we have discussed the couplings between adjacent joints. To probe the overall behavior of the arm consisting of several joints, one should take the multivariate nature of the time series into account. Recently, various studies have been conducted, unveiling dynamic characteristics of multivariate time series [43–47]. With the focus on the dynamic coupled behavior, we perform the multiscale network analysis [43] using the isolated time series. To be specific, we compose three multivariate time series consisting of four isolated time series of each component (e.g., Δ*r*_*X*_, Δ*f*_*X*_, Δ*w*_*X*_, and Δ*e*_*X*_ in the case of the *X*-component) and compute the clustering coefficient entropy *E*_*c*_ of the inferred network from each multivariate series. The results of the *Z*-component are shown in Fig 6. Notably, the difference between the normal group and the patient group is statistically significant on every scale. Exactly the same results are also observed in *X*- and *Y*-components, confirming the enhanced coupled behavior in the patient group.

#### Phase Synchrony and Granger Causality between Components

As the final outcome of the coupling between joints, the arm-posture dynamics is realized as the time series of the rod end. We may then elucidate the underlying structure of the emergent dynamics by probing the coupling between components, e.g., between *X*- and *Y*-components, of the rod time series $s_{\alpha }^{1}\equiv r_{\alpha }$ (bare one rather than the isolated one Δ*r*_*α*_). In this direction, we also evaluate the original phase synchrony Ψ(*r*_*α*_, *r*_*β*_) and Granger causality *G*_*r*_*α*_ → *r*_*β*__ rather than isolated measures, where *r*_*β*_ is one of the *X*-, *Y*- and *Z*-components of the rod time series (obviously *β* ≠ *α*). Note that the dynamics of each component is accomplished in a different manner: Only *X*- and *Z*-components are directly related to the performance while the *Y*-component remains free from the task. Further, among *X*- and *Z*-components, only the *Z*-component is subject to the external gravitational force. The results are summarized in Table 3.

**Table 3:** Coupling/locking relations between components.

	Normal	Patient		Normal	Patient
Ψ_1_(*r*_*X*_, *r*_*Y*_)	0.30 ± 0.07	0.59 ± 0.17*	*G*_*r*_*X*_ → *r*_*Y*__	0.051 ± 0.039	0.070 ± 0.040
Ψ_1_(*r*_*Y*_, *r*_*Z*_)	0.088 ± 0.057	0.12 ± 0.06	*G*_*r*_*Y*_ → *r*_*X*__	0.28 ± 0.06	0.14 ± 0.06*
Ψ_1_(*r*_*Z*_, *r*_*X*_)	0.19 ± 0.05	0.23 ± 0.08	*G*_*r*_*Y*_ → *r*_*Z*__	0.36 ± 0.18	0.16 ± 0.08
Ψ_2_(*r*_*X*_, *r*_*Y*_)	0.087 ± 0.032	0.39 ± 0.22*	*G*_*r*_*Z*_ → *r*_*Y*__	0.11 ± 0.06	0.13 ± 0.04
Ψ_2_(*r*_*Y*_, *r*_*Z*_)	0.32 ± 0.13	0.23 ± 0.15	*G*_*r*_*Z*_ → *r*_*X*__	0.022 ± 0.012	0.043 ± 0.019
Ψ_2_(*r*_*Z*_, *r*_*X*_)	0.082 ± 0.033	0.14 ± 0.04*	*G*_*r*_*X*_ → *r*_*Z*__	0.082 ± 0.051	0.057 ± 0.046

Phase synchrony and Granger causality between the three components. Asterisks (*) denote statistically significant normal-patient group pairs (student’s *t*-test, *p* < 0.05). Compared with the normal group, phase synchrony is stronger while Granger causality is weaker in the patient group which indicates ‘locked’ relations between the components in patients.

We first analyze the phase synchrony to identify the coupling strength between components. Comparing the order parameter Ψ_1_, we find that patients have significantly stronger couplings between *X*- and *Y*-components than normal subjects. Similarly, comparison of the order parameter Ψ_2_ manifests that the synchrony between *X*- and *Z*-components is stronger in the patient group as well. It is thus confirmed primarily that the coupling between components is stronger in the patient group.

Next, we examine the Granger causality between components. First, let us determine the causal relation between the components by comparing *G*_*r*_*α*_ → *r*_*β*__ and *G*_*r*_*β*_ → *r*_*α*__. For example, in the case of the *X*-*Y* pair of normal subjects, *G*_*r*_*Y*_ → *r*_*X*__ is significantly larger than *G*_*r*_*X*_ → *r*_*Y*__ (student’s *t*-test *p* < 0.05), which demonstrates that *Y*-component dynamics is the Granger cause of *X*-component dynamics. In this way, we can determine the direction of all three pairs for normal subjects: *r*_*Y*_ is the cause of *r*_*X*_ and *r*_*Z*_ whereas *r*_*X*_ is the cause of *r*_*Z*_. Note that the Granger causality between *X*- and *Z*-components is rather small. This observation is also true in phase synchrony as well [Ψ_2_(*r*_*Z*_, *r*_*X*_) is small in the normal group as discussed above], indicating that *X*- and *Z*-component dynamics are decoupled in the normal group.

In the case of patients, on the other hand, Granger causality does not reveal any statistically significant pairs. In consequence, we fail to determine the directions of couplings. Moreover, the Granger causality is even weaker than the normal group (namely, the difference in *G*_*r*_*Y*_ → *r*_*X*__ is statistically significant), which appears contradictory to the phase synchrony result (as Ψ_1_(*w*_*Y*_) and *G*(*w*_*Y*_) of the patient group in the previous section). To resolve this contradiction, we inspect the time series closely and conclude that the *X*- and the *Y*-components of the time series are very similar to each other in the patient group. We also check that the *Y*- and the *Z*-components are very similar as well if we take into account the fact that the overall sign of the *Z*-component is irrelevant to computing Ψ_2_ (albeit weaker than the *X*-*Y* pair). In this case, the Granger causality could have small values because the information flow between the time series is negligible. Still, the phase synchrony could be large due to the similarity itself between the time series. Therefore, it would be more appropriate to call the relation between the components ‘locked’ rather than ‘coupled’. On the other hand, we cannot find any simple pattern in the inspection of the bare time series of normal subjects.

#### Response Mechanism of Normal Subjects and Reduction of the Dynamic Degrees of Freedom in Patients

We are now ready to describe the underlying mechanism of the arm-posture dynamics. We stress here that the *Y* component plays a significant role in the coupled dynamics of the system. As already stated, *Y* component dynamics is the cause of the dynamics of *X*- and *Z*-components in the normal subject group. Also as one can confirm in Table 3, phase synchrony of *Y*-*X* and *Y*-*Z* pairs is weaker than that in patients but relatively large if compared with that of the *X*-*Z* pair. This unique feature of the *Y* component can be interpreted as a consequence of the external gravitational force. Because the subject sustains the arm and the rod against the gravity, fatigue is accumulated as time proceeds. Due to the task goal to maintain the position of the rod, the subject should adopt an indirect strategy against the gravitational force: shrinking the arm to reduce the torque acting on the arm rather than dropping the arm. As a result, the *Y*-component time series which is left free from the task displays an overall decreasing tendency. These decreasing patterns in the *Y*-direction while sustaining in the *X*- and *Z*-directions are actually accomplished by folding joints. To compensate the fluctuations originating from the *Y*-component dynamics, the *X*- and the *Z*-components should respond and accordingly, causal relations are observed in the case of *Y*-*X* and *Y*-*Z* pairs. Even though the decreasing pattern itself cannot be observed in the high-pass filtered time series, we conclude that the underlying perturbation-response mechanism remains in the time series.

**Fig 7:**
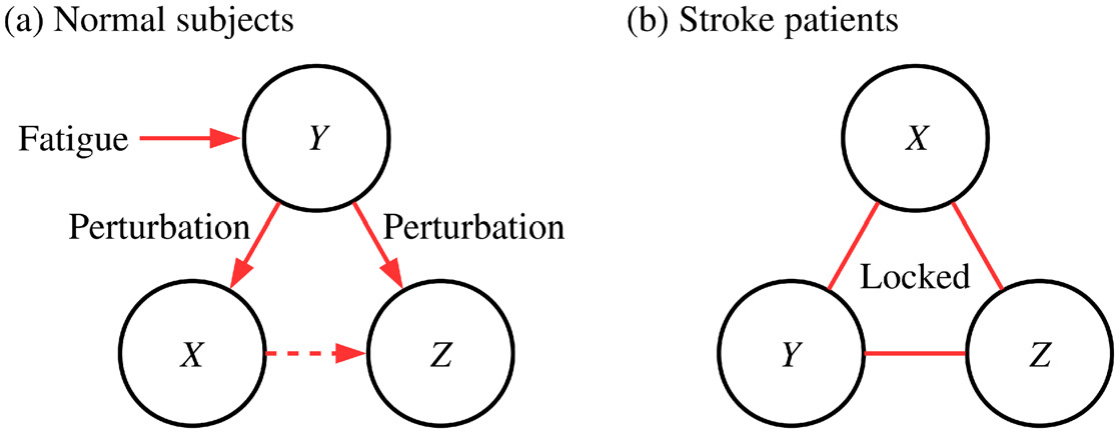
Schematic diagrams of the arm-posture dynamics. Shown in (a) and (b) are schematic diagrams describing the conceptual coupling structures of normal subjects and stroke patients, respectively.

Such patterns are deteriorated, however, in the case of the patients because the components are locked together. In other words, overall phase synchrony of components is strong while Granger causality remaining small, because the three time series actually carry similar information. We conclude that the rigidity of the system causes the reduction of degrees of freedom, namely, the loss of dynamic components. The coupling relationship and the response mechanism are illustrated schematically in Fig 7a and 7b.

Here one should be careful about the limitation inherent in the pairwise analysis. Specifically, the direct coupling from the *Y*-component to the *Z*-component in the normal group is not distinguishable from the indirect coupling via the *X*-component. In fact, the coupling between *X*- and *Z*-components is weak as indicated by the dotted arrow; accordingly, it is expected that the effects via the *X*-component are small. For more clarity, we examine the multivariate Granger causality measure from the *Y*- to *Z*-component, conditioned on the *X*-component time series. Obtained values of *G*_*r*_*Y*_ → *r*_*Z*_∣*r*_*X*__ and *G*_*r*_*Z*_ → *r*_*Y*_∣*r*_*X*__ are 0.32 ± 0.15 and 0.11 ± 0.07, respectively, with the difference being statistically significant (student’s *t*-test, *p* < 0.05). This confirms that the conclusion deduced from the pairwise analysis is indeed valid.

## Summary and Perspectives

Analyzing the time series of normal subjects and stroke patients, we have recognized three unique features of the goal-directed arm-posture dynamics. First, we have observed that the dynamics of normal subjects is not periodic at all, as confirmed by the overall power-law behavior of the power spectrum, while the oscillatory behavior of pathological tremors, identified as low-dimensional chaos, emerges in the dynamics of patients. Second, we have quantified that couplings between the joints of patients are stronger than those of normal subjects. Third, we have addressed the way how the human arm responds to the gravitational force, which emphasizes the essential role of *Y*-component dynamics. Interestingly, totally different measures have turned out to give consistent results, illuminating various aspects of the dynamical complexity.

We have also proposed a theoretical framework describing the arm-posture dynamics. In the case of the normal subject group, joints and components are coupled appropriately to respond to external perturbations such as the gravitational force. As fatigue is accumulated due to the gravity, the individual shrinks the arm to minimize the torque and then adjusts finely positions of the joints to perform the task. On the other hand, the dynamical components are overly coupled in the patient group. These locked features cause the reduction of the degrees of freedom and the emergence of 2 to 3 Hz tremors, which obscure the responses of the patients to the environments.

Based on these findings, we now suggest that the normal arm-posture dynamics and the loss of complexity representing the patient dynamics are emergent phenomena from the couplings between joints. As well known in the field of statistical physics, the coupling strength is the key ingredient tuning collective properties of a macroscopic system. In this perspective, we conclude that the dynamics and loss of complexity of patients is a consequence of the macroscopic order arising from the pathologically strong couplings between joints. On the other hand, the dynamics of normal subjects does not involve the ordered state, as manifested by the lack of peaks in the power spectrum.

It is of interest to note the unusual property of the *Y*-component time series which plays a crucial role in the response mechanism. On one hand, we observe the 1/*f* ^2^ power spectrum which is characteristic of Brownian noise. On the other hand, every *Y*-component time series of normal subjects (except #1 encountering the technical problem) passes the surrogate test, providing a quantitative evidence of the inherent nonlinearity in the dynamics (not discussed in this paper). Provisionally, we conclude that *Y*-component dynamics reflects neither low-dimensional chaos nor simple Brownian noise. Detailed analysis should illuminate the novel feature of the *Y*-component time series. Its relation to the essential role in the dynamics, as well as mathematical modeling and additional experiments for quantitative verification, is left for further study.

Finally, we point out that there are several limitations in this work. First of all, the number of subjects is somewhat small. Even though crucial differences between the normal group and the patient group are already manifested unambiguously, analysis and comparison of larger data would be of more benefit to revealing detailed structure. Second, the analysis suffers from the shortage of the time series. In our experience, longer time series are apt to suffer from non-stationarity because fatigue could affect the *Z*-component dynamics rather directly. Nevertheless, it could be possible to modify the experimental setup and to delay such fatigue effects.

## Materials and Methods

### Subjects

Six stroke patients and six normal subjects participated in the experiment. Participants were recruited through the local senior center. Written informed consent of all participants was obtained. All procedures involving human participants were approved by the University Institutional Review Board (Seoul National University, IRB No. 0806/001-001). Inclusion criteria for stroke patients in the study consisted of the following: (1) absence of other neurological deficits; (2) currently not participating in another upper extremity rehabilitation program; (3) diagnosis of chronic states having strokes more than 12 months ago; (4) cognitive competency to give informed consent and to understand and follow practitioner’s instructions.

### Upper-limb Posture Control Assessment

The experimental task was goal-directed arm posture, to sustain a rod pointing to a target in front of the subject. Each subject performed the task as stable as possible for 30 seconds. Positions of the end point of the rod and joints of the subject were measured. Kinematic data were collected by an optoelectronic motion capture system (Qualisys) with eight CCD cameras. Spherical markers were placed on well-recognizable anatomical landmarks: on the shoulder, over the lateral border of the acromion; on the upper arm in proximity of the elbow, over the lateral humeral epicondyle; on the forearm in proximity of the wrist, on the styloid process of the ulna; and on the hand dorsum, on the head of the second metacarpal, which are dubbed shoulder, elbow, wrist, and finger joints, respectively, in this paper. Two more markers were placed at the end of the rod and at the target. For each subject, 20 second time series was acquired with a sampling rate of 100 Hz and stored for off-line analysis.

### Non-linear Time Series Analysis

#### Takens Time Delay Embedding

The first step of the nonlinear time series analysis is to embed the time series in an *m*-dimensional phase space and to reconstruct the attractor of the dynamics. Here we employ the Takens time delay embedding [35]. Such embedding was shown mathematically to be possible in the case of the infinite-length time series for *m* > (2*d* + 1) where *d* is the actual dimension of the attractor of the dynamical system. For time series {*s*(1), *s*(2), ⋯, *s*(*N*)}, the delay embedding is performed as follows:

$$\begin{array}{c} \vec{s}\left(t\right)=\left(s\left(t-(m-1)\tau \right),s\left(t-(m-2)\tau \right),\cdots ,s\left(t\right)\right), \end{array}$$ (1)

where *τ* is the time delay. Specifically, time delay *τ* = 5 and embedding dimension *m* = 10, 12, ⋯, 26 have been used in this paper.

#### Dimensional Complexity

The revised correlation sum *C*(*ϵ*) is defined to be [33, 36]:

$$\begin{array}{c} C\left(\epsilon \right)=\frac{2}{\left(N-W)(N-1-W\right)}\sum_{t_{1}=1}^{N}\sum_{t_{2}=t_{1}+1+W}^{N}\theta (\epsilon -||\vec{s}\left(t_{1}\right)-\vec{s}\left(t_{2}\right)||), \end{array}$$ (2)

where *W* denotes the Theiler correction and ∣∣⋯∣∣ the appropriate distance in the *m*-dimensional space. In this paper, we have taken *W* = 5 and $\left|\right|\vec{s}\left(t_{1}\right)-\vec{s}\left(t_{2}\right)\left|\right|\equiv \text{max}\{|s\left(t_{1}-k\tau \right)-s\left(t_{2}-k\tau \right)\left|:0\leq k\leq m-1\right\}$. The dimensional complexity is then given by

$$\begin{array}{c} D_{2}=\lim_{\epsilon \to 0}\frac{\partial \ln C(\epsilon )}{\partial \ln\epsilon }. \end{array}$$ (3)

Technically, if a plateau is found in the flow of ∂ ln *C*(*ϵ*)/∂ ln *ϵ* plotted versus ln *ε*, the value at the plateau is identified as the correlation dimension *D*_2_ of the attractor.

#### Multi-scale Sample Entropy

The entropy is a traditional measure of the nonlinear dynamics theory, elucidating regularity of a time series. We begin with the Reyni entropy [48]:

$$\begin{array}{c} K_{q}=\lim_{\tau \to 0}\lim_{\epsilon \to 0}\lim_{m\to \infty }\frac{1}{m\tau }\frac{1}{1-q}\ln\sum_{i_{1},\cdots ,i_{m}}{\left[p(i_{1},\cdots ,i_{m})\right]}^{q} \end{array}$$ (4)

Noting Eq (2), one obtains the relation [49]

$$\begin{array}{c} C\left(\epsilon \right)∼\sum_{i_{1},\cdots ,i_{m}}{\left[p(i_{1},\cdots ,i_{m})\right]}^{2}. \end{array}$$ (5)

With the aid of Eq (5), we can calculate the Reyni entropy of order two or the Grassberger-Procaccia entropy, which provides a lower bound of the Kolmogorov-Sinai entropy, in terms of the correlation sum [36]:

$$\begin{array}{c} K_{2}=\lim_{m\to \infty }\lim_{\epsilon \to 0}K_{2,m}\left(\epsilon \right)=\lim_{m\to \infty }\lim_{\epsilon \to 0}\frac{1}{\tau }\ln\frac{C_{m}\left(\epsilon \right)}{C_{m+1}\left(\epsilon \right)}. \end{array}$$ (6)

In general it is easier to compute *K*_2_ from the time series than the Kolmogorov-Sinai entropy, and the method described above is favored in the time series analysis.

In the case of an experimentally measured time series, however, the limit process in the definition of the entropy is not possible due to the limitation on the length of the time series. Therefore, finite values of *m* and non-zero values of *ϵ* are used in practice. Based on the sample entropy [50], one may introduce the multiscale entropy *S*_*E*_[34] by defining the re-scaled time series

$$\begin{array}{c} y^{\left(\tau \right)}\left(t\right)\equiv \frac{1}{\tau }\sum_{t=(t^{\prime }-1)\tau +1}^{t^{\prime }\tau }s\left(t\right). \end{array}$$ (7)

As the scale factor *τ* is increased, components with short autocorrelations, which are usually regarded as noise, become reduced. Therefore irregularity originating from randomness can be eliminated, exposing bona fide complexity.

### Phase Synchrony

To isolate dynamics of a single joint from that of other joints, we first extract the difference of the time series between adjacent joints in the following way:

$$\begin{array}{c} \Delta s_{\alpha }^{i}\left(t\right)\equiv s_{\alpha }^{i-1}\left(t\right)-s_{\alpha }^{i}\left(t\right). \end{array}$$ (8)

Note that the isolated dynamics of joint *i* is realized by the difference between its own time series $s_{\alpha }^{i}$ and the time series $s_{\alpha }^{i-1}$ of the following joint *i* − 1. In this way, we extract dynamics of each detached joint, eliminating the effects of its precedent joint. In particular, the phase synchrony between the series $\Delta s_{\alpha }^{i}$ and $\Delta s_{\alpha }^{i+1}$ allows one to analyze whether dynamics of a certain joint is free from dynamics of its precedent joint. To obtain the phase synchrony between joints (see Table 2), we first calculate the phase $\phi_{\alpha }^{i}\left(t\right)$ of the time series from the analytic signal $\zeta_{\alpha }^{i}\left(t\right)\equiv \Delta s_{\alpha }^{i}\left(t\right)+is_{\alpha }^{i,H}\left(t\right)\equiv A_{\alpha }^{i}\left(t\right)\;e^{i\phi_{\alpha }^{i}\left(t\right)}$, where $s_{\alpha }^{i,H}\left(t\right)$ is given by the Hilbert transform

$$\begin{array}{c} s_{\alpha }^{i,H}\left(t\right)=\frac{1}{\pi }P\int_{-\infty }^{\infty }\frac{\Delta s_{\alpha }^{i}\left(\tau \right)}{t-\tau }d\tau \end{array}$$ (9)

with P denotes the principal value. Finally, we define the order parameters $\Psi_{1}\left(s_{\alpha }^{i}\right)$ and $\Psi_{2}\left(s_{\alpha }^{i}\right)$ representing dynamic originality of joint *i* in the *α*-direction:

$$\Psi_{1}\left(\Delta s_{\alpha }^{i},\Delta s_{\alpha }^{i+1}\right)e^{i\Phi_{1}\left(\Delta s_{\alpha }^{i},\Delta s_{\alpha }^{i+1}\right)}=\frac{1}{N}\overset{N}{\underset{t=1}{\sum }}e^{i\left(\phi_{\alpha }^{i}-\phi_{\alpha }^{i+1}\right)}$$ (10)

$$\Psi_{2}\left(\Delta s_{\alpha }^{i},\Delta s_{\alpha }^{i+1}\right)e^{i\Phi_{2}\left(\Delta s_{\alpha }^{i},\Delta s_{\alpha }^{i+1}\right)}=\frac{1}{N}\overset{N}{\underset{t=1}{\sum }}e^{2i\left(\phi_{\alpha }^{i}-\phi_{\alpha }^{i+1}\right)},$$ (11)

which measure the conventional in-phase synchrony and the out-of-phase synchrony, respectively.

On the other hand, we use the bare time series of the rod end to compute phase synchrony among the *X*-, *Y*- and *Z*-components (Table 3). In this case, we calculate the phase *θ*_*α*_(*t*) of the series *r*_*α*_(*t*) from the analytic signal $\zeta_{\alpha }\left(t\right)\equiv r_{\alpha }\left(t\right)+ir_{\alpha }^{H}\left(t\right)\equiv A_{\alpha }\left(t\right)e^{i\theta_{\alpha }\left(t\right)}$ with the Hilbert transform

$$\begin{array}{c} r_{\alpha }^{H}\left(t\right)=\frac{1}{\pi }P\int_{-\infty }^{\infty }\frac{r_{\alpha }\left(\tau \right)}{t-\tau }d\tau \end{array}$$ (12)

and compute the order parameters

$$\Psi_{1}\left(r_{\alpha },r_{\beta }\right)e^{i\Phi_{1}\left(r_{\alpha },r_{\beta }\right)}=\frac{1}{N}\overset{N}{\underset{t=1}{\sum }}e^{i\left(\theta_{\alpha }-\theta_{\beta }\right)}$$ (13)

$$\Psi_{2}\left(r_{\alpha },r_{\beta }\right)e^{i\Phi_{2}\left(r_{\alpha },r_{\beta }\right)}=\frac{1}{N}\overset{N}{\underset{t=1}{\sum }}e^{2i\left(\theta_{\alpha }-\theta_{\beta }\right)}.$$ (14)

### Granger Causality

For two (arbitrary) series *u* and *v*, the regression

$$\begin{array}{c} u\left(t\right)=\sum_{k=1}^{q}A_{u,k}u\left(t-k\right)+\sum_{k=1}^{q}A_{v,k}v\left(t\right)+\eta_{u,t}, \end{array}$$ (15)

and the reduced regression

$$\begin{array}{c} u\left(t\right)=\sum_{k=1}^{q}B_{u,k}u\left(t-k\right)+\xi_{u,t} \end{array}$$ (16)

are carried out, where *q* is determined by the Akaike information criterion [42]. Then the Granger causality *G*_*v* → *u*_ from time serise *v*(*t*) to *u*(*t*) is defined as [42]

$$\begin{array}{c} G_{v\to u}\equiv \ln\frac{\Sigma_{\xi }}{\Sigma_{\eta }} \end{array}$$ (17)

where Σ_*η*_ = cov(*η*_*u*, *t*_) and Σ_*ξ*_ = cov(*ξ*_*u*, *t*_) are the residuals covariances of the regression models. To probe the relation between the joints, we compute the isolated causality measure $G_{\Delta s_{\alpha }^{i+1}\to \Delta s_{\alpha }^{i}}$ using the isolated time series $\Delta s_{\alpha }^{i}\left(t\right)$ and $\Delta s_{\alpha }^{i+1}\left(t\right)$, as shown in Table 2. On the other hand, the coupling/locking relations between components are measured by the Granger causality *G*_*r*_*α*_ → *r*_*β*__ computed from the bare time series of the rod end (see Table 3).

## Supporting Information

S1 File Complete time series data. Columns A, B and C display 2000 data points (corresponding to 20 seconds), respectively, of the *X*-, *Y*- and *Z*-component time series of the rod end. Similarly, columns D, E, F / G, H, I / J, K, L / M, N, O tabulate 2000 data points of the *X*-, *Y*-, *Z*-component time series of the finger / wrist / elbow / shoulder, respectively.
(XLSX)
